# Cholinergic signaling influences the expression of immune checkpoint inhibitors, PD-L1 and PD-L2, and tumor hallmarks in human colorectal cancer tissues and cell lines

**DOI:** 10.1186/s12885-023-11410-3

**Published:** 2023-10-12

**Authors:** Nyanbol Kuol, Janusz Godlewski, Zbigniew Kmiec, Sara Vogrin, Sarah Fraser, Vasso Apostolopoulos, Kulmira Nurgali

**Affiliations:** 1 Institute for Health and Sport, Victoria University, Melbourne, Australia; 2 Department of Physiology and Cell Biology, University of Nevada, Reno, USA; 3 University of Warmia and Mazury, Olsztyn, Poland; 4 Department of Histology, Medical University of Gdansk, Gdansk, Poland; 5 Department of Medicine Western Health, University of Melbourne, Melbourne, Australia; 6 Immunology Program, Australian Institute of Musculoskeletal Sciences, Melbourne, Australia; 7 Regenerative Medicine Program, Australian Institute of Musculoskeletal Sciences, Melbourne, Australia

**Keywords:** CRC, Immunosuppressive, PD-L1, PD-L2, Cholinergic, M3R, α7nAChR, ChAT

## Abstract

**Background:**

Cancer cells express immunosuppressive molecules, such as programmed death ligands (PD-L)1 and PD-L2, enabling evasion from the host’s immune system. Cancer cells synthesize and secrete acetylcholine (ACh), acting as an autocrine or paracrine hormone to promote their proliferation, differentiation, and migration.

**Methods:**

We correlated the expression of PD-L1, PD-L2, cholinergic muscarinic receptor 3 (M3R), alpha 7 nicotinic receptor (α7nAChR), and choline acetyltransferase (ChAT) in colorectal cancer (CRC) tissues with the stage of disease, gender, age, risk, and patient survival. The effects of a muscarinic receptor blocker, atropine, and a selective M3R blocker, 4-DAMP, on the expression of immunosuppressive and cholinergic markers were evaluated in human CRC (LIM-2405, HT-29) cells.

**Results:**

Increased expression of PD-L1, M3R, and ChAT at stages III-IV was associated with a high risk of CRC and poor survival outcomes independent of patients’ gender and age. α7nAChR and PD-L2 were not changed at any CRC stages. Atropine and 4-DAMP suppressed the proliferation and migration of human CRC cells, induced apoptosis, and decreased PD-L1, PD-L2, and M3R expression in CRC cells via inhibition of EGFR and phosphorylation of ERK.

**Conclusions:**

The expression of immunosuppressive and cholinergic markers may increase the risk of recurrence of CRC. These markers might be used in determining prognosis and treatment regimens for CRC patients. Blocking cholinergic signaling may be a potential therapeutic for CRC through anti-proliferation and anti-migration via inhibition of EGFR and phosphorylation of ERK. These effects allow the immune system to recognize and eliminate cancer cells.

**Supplementary Information:**

The online version contains supplementary material available at 10.1186/s12885-023-11410-3.

## Background

Colorectal cancer (CRC) is the third leading cause of cancer-related death worldwide due to a predominantly unhealthy lifestyle and genetic factors [1]. CRC presents vague or no symptoms at the early stages; hence, it is more often diagnosed at the advanced stages of a disease. About 70% of CRC occurs sporadically due to the accumulation of mutations in the tumor suppressor genes that induce cancer [2], such as *p53* and *adenomatous polyposis coli* [3, 4]. However, studies have demonstrated that immunosuppression and cholinergic signaling play an essential role in developing and progressing CRC [5–10].

The immune system plays a pivotal role in the development of tumors. It not only suppresses growth but can also advance tumor growth by creating an immunosuppressive environment. The ability of cancer cells to evade T cell responses and avoid immune recognition by disabling effector T cells is dependent on the multiple immunosuppressive mechanisms controlled by immune checkpoints of inhibitory pathways, including, but not limited to, programmed death-ligand (PD-L)1 and PD-L2. Ligand-receptor interactions initiate these immune checkpoints to enhance anti-tumor immunity [11]. Cancer cells overexpress immunosuppressive factors, such as PD-L1 and PD-L2, leading to suppressed T cell activation and apoptosis [10]. PD-L1 is a transmembrane protein that plays a significant role in suppressing the immune system. Several cancer cells and antigen-presenting cells express PD-L1 [12]. T cells express the receptor programmed death-1 (PD-1); upon interaction with PD-L1, inhibitory signals are triggered, resulting in reduced activation or exhaustion of CD8 + T cells [13]. Similarly, PD-L2 interacts with PD-1 on activated CD8 + T cells. However, its role in maintaining tumor cell immunity is unclear [14]. These findings have suggested the importance of the interaction between the tumor and the host’s immune system, allowing cancer cells to evade the immune system leading to growth and spread.

The nervous system also plays a functional role in cancer growth and progression. Studies have demonstrated that the nervous system promotes cancer cell development by facilitating angiogenesis and metastasis by releasing neural-related factors from nerve endings such as neurotrophins, neuropeptides, and neurotransmitters [6, 7]. One of the major neurotransmitters in the central and peripheral nervous system is acetylcholine (ACh), which can cause diverse effects depending on the receptor type. Recently, studies have shown that several cancers can release ACh and express cholinergic receptors, suggesting that ACh could play a significant role in tumor growth, vascularization, invasion, and metastasis [15, 16]. ACh has been shown to promote the proliferation and migration of cancer cells, angiogenesis, and metastasis through the activation of muscarinic receptor 3 (M3R) and alpha 7 nicotinic receptor (α7nAChR) [17–20]. In addition, cancer cells can also overexpress choline acetyltransferase (ChAT), a precursor enzyme required for ACh synthesis [18].

No studies have correlated the expression of immunosuppressive and cholinergic markers with CRC stages and clinical parameters. It is necessary to identify the combination of factors expressed by cancer cells that may be important for early cancer detection and may predict cancer progression in patients. Therefore, in this study, the expression of immunosuppressive (PD-L1 and PD-L2) and cholinergic (M3R, α7nAChR, and ChAT) markers was correlated with CRC stages (I-IV), patients’ age, gender, risk, and survival outcomes. In addition, the mechanisms involved in the effects of blocking cholinergic signaling on the expression of immunosuppressive molecules were determined in vitro.

## Methods

### Human cancer samples

Colorectal cancer tissues were collected at the Ministry of Internal Affairs and Administration Hospital in Olsztyn, Poland, from 2010 to 2013. The Bioethics Committee of the University of Warmia and Mazury in Olsztyn, Poland (decision no. 3/2010 and 34/2010), approved the collection of human samples used in this study. The study comprised 91 patients with CRC (with clinical follow-up). All patients signed written informed consent to use their tissues for research purposes. Patients had no evidence of bowel obstruction or other colonic diseases. None of the CRC patients had a second neoplastic disease or had previously undergone chemo- or radiotherapy. Patients’ demographical, clinical, and overall survival data were collected. The type of cancer and grading were described by a pathologist according to the World Health Organization criteria and staging according to the 7th edition of the Cancer Staging Manual of the American Joint Committee on Cancer (AJCC). However, due to a small patient number with grade III (n = 7) compared to grade II (n = 84) CRC and the low number of patients with metastases (n = 9) compared to non-metastatic disease (n = 82), grade and metastasis were excluded from the multivariate analysis and Chi-Square test. Stages I and II were grouped, and stages III and IV were combined into another group. Tumor tissues were collected into 10% neutral buffered formalin, dehydrated in ethanol/xylene, and embedded in paraffin wax. Paraffin-embedded tissue blocks were cut into 4 μm thickness sections and mounted onto the microscope slides.

### Cell culture

Human colon cancer cell line LIM-2405 was obtained from the Ludwig Institute for Cancer Research (CCBA-0165, Melbourne, Australia), and human colon adenocarcinoma cell line HT-29 was obtained from the American Tissue Culture Collection (HTB-38, ATCC® VA, USA). LIM-2405 and HT-29 were cultured in Roswell Park Memorial Institute (RPMI) 1640 medium (Sigma-Aldrich, Castle Hill, Australia). Culture media was supplemented with 10% fetal bovine serum, 1% penicillin-streptomycin, and 1% glutamine. Cells were cultured at 37 °C, 5% CO_2_, and 95% air atmosphere. When cells grew into confluent or semiconfluent monolayers in 75 cm^2^ medium flasks, they were either passaged or used. The passage of cells was conducted with 0.25% trypsin every 3–4 days.

### Cell proliferation

The water-soluble tetrazolium-1 (WST-1) assay kit (Roche Diagnostics GmbH, Germany) was used to determine the viability of LIM-2405 and HT-29 cells. WST-1 is cleaved to form formazan dye via a complex cellular interaction at the cell surface. This interaction is contingent on the viable cells’ production of glycolytic nicotinamide adenine dinucleotide phosphate (NADPH). Hence, the amount of formed formazan dye correlates with the number of viable cells in the culture. LIM-2405 and HT-29 cells were seeded and cultured at 1 × 10^4^ cells per well in 96-well plates for 24 h (hrs). Cells were then treated with various concentrations of the general muscarinic receptor blocker, atropine (Sigma-Aldrich, Australia) for 1–48 h, selective M3R blocker, 1,1-dimethyl-4-diphenylacetoxypiperidinium iodide (4-DAMP) (Abcam, Australia), cholinergic agonist, carbachol (Abcam, Australia) and acetylcholinesterase inhibitor, donepezil (Abcam, Australia) for 8 h. All treatments were performed in triplicates, and three independent experiments were conducted. WST-1 reagent (10 µL) was added to each well and incubated at 37 °C for 1 h. Cellular proliferation at the absorbance of 450 nm was measured using a microplate reader (Varioskan Flash, Thermo Scientific, Australia).

### Migration assay

LIM-2405 and HT-29 cell lines were used in migration analysis using Boyden chambers with 8 μm pore size membrane filter inserts (Corning Costar Corp., Kennebunk, ME, USA) in 24 well tissue culture plates. The cells were trypsinized and resuspended in serum-free RPMI-1640 media at 2 × 10^5^ cells per mL density. A total of 200 µL of cell suspension was seeded in the Transwells’ upper chamber and 600 µL of media into the lower chamber. Cells were treated with 0 and 100 μm atropine (Sigma-Aldrich, Melbourne, Australia) for 8, 24, and 48 h. The chambers were incubated at 37 °C in a 5% CO_2_ incubator. After 8–48 h, the non-migrating cells on the upper surface of the insert were removed, and cells that migrated to the underside of the membrane were counted using a light microscope. In all experiments, two independent experiments were conducted in duplicates.

### Annexin V apoptosis assay

LIM-2405 and HT-29 cell lines were cultured overnight in six wells at the density of 5 × 10^5^ cells per well. Cells were treated with 100 µM atropine and 4-DAMP for 8 h. Following treatments, flow cytometry was utilized to determine the apoptotic and necrotic cells. Cells were collected and resuspended in fluorescence-activated cell sorting (FACS) buffer and labeled 100 µL per well with Annexin V at 1:1,000 dilution and 0.5 µg/mL of propidium iodide (PI). In all experiments, two independent experiments were conducted in duplicates.

### Choline/acetylcholine assay

The choline/acetylcholine assay kit (Abcam, Australia) was used to measure choline concentration in LIM-2405 and HT-29 cell lysates. The assay was carried out per the instructions provided by the manufacturer. Briefly, LIM-2405 and HT-29 (1 × 10^6^) cells were cultured overnight, after which cells were treated with 100 µM of cholinergic antagonists, atropine, and 4-DAMP and 300 µM of acetylcholinesterase inhibitor, donepezil. Cells were lysed in 500 µL choline assay buffer before commencing choline measurements using a microplate reader (Varioskan Flash, Thermo Scientific, Australia) at the absorbance of 570 nm. All treatments were performed in duplicates, and two independent experiments were conducted.

### Immunofluorescence and immunocytochemistry staining

Paraffin-embedded tissue sections were deparaffinized and hydrated through a series of washes with xylene and graded alcohol. Antigen retrieval was performed using citrate buffer pH 6.0, 10x (Sigma-Aldrich, Melbourne, Australia). The citrate buffer was heated until bubbles started to form. Samples were immersed into the buffer, placed on a hot plate pre-set at 100 °C for 15 min (mins), and left to cool at room temperature for another 20 min. LIM-2405 and HT-29 cells were grown on chamber slides (Ibidi, Australia) overnight at 37 °C in a 5% CO_2_ incubator. Cells at a density of 1 × 10^5^ cells per well were treated with atropine (0 and 100 µM) for 8 h and fixed in 4% paraformaldehyde for 10 min. Cells were permeabilized for 15 min in 0.1% phosphate-buffered saline (PBS). The endogenous activities in human samples and cell lines were blocked using 10% donkey serum for 1 h at room temperature. Human CRC tissues and cell lines were incubated overnight at room temperature with 1:500 dilution of primary antibodies, [mouse monoclonal to PD-L1 (Abcam, ab210931), rabbit polyclonal to PD-L2 (Abcam, ab200377), rabbit polyclonal to M3R (Abcam, ab126168), mouse monoclonal to α7nAChR (Novus, 7F10G1) and goat polyclonal to ChAT (ab134021)]. After washing in PBS plus Triton X-100 (PBST), samples were incubated at room temperature for 2 h with 1:250 dilution of secondary antibodies [Alexa Fluor 647-conjugated donkey anti-mouse (Abacus, JI715605150), Alexa Fluor 594-conjugated donkey anti-rabbit (Abacus, JI711585152), and Alexa Fluor 488-conjugated donkey anti-goat and anti-mouse (Abacus, JI705-545-003)] diluted in PBS containing 2% donkey serum and 0.01% Triton X-100. Samples were then incubated for 1 min with 4′,6-diamidine-2′-phenylindole dihydrochloride (DAPI) (D1306, Life Technologies, Australia) and mounted with DAKO mounting medium (Agilent Technologies, Australia). Coverslips were placed onto slides, and samples were left to dry overnight before imaging.

### Western blot

Expression of immunosuppressive and cholinergic markers, as well as cell signaling pathways in LIM-2405 and HT-29 cell lines, were evaluated by western blot using mouse monoclonal antibody to pSTAT3 (Cell Signalling, Australia, #9145), rabbit monoclonal antibody to pERK1/2 (Cell Signalling, Australia, #3192), and rabbit monoclonal antibody to EGFR (Cell Signalling, Australia, #4267)) at 1:1000 dilution. Cells were incubated with 100 µM atropine and 4-DAMP for 8 h. After treatments, cells were collected and lysed in radioimmunoprecipitation assay (RIPA) buffer (pH 7.4, 150 mM NaCl, 0.1% sodium dodecyl sulfate (SDS), 0.5% sodium deoxycholate, 1% NP-40 in PBS, Sigma) containing a protease and phosphatase inhibitors cocktail (Roche Applied Science). Cellular proteins (20 µg) from each sample were separated by 8–12% SDS/polyacrylamide gel electrophoresis. The separated fragments were transferred to 0.22 μm polyvinylidene fluoride membranes, blocked with 5% skim milk in PBS containing 0.1% Tween 20 overnight at 4 °C at 40 revolutions per minute (RPM) speed shaker. The membranes were incubated with primary antibodies overnight at 4 °C, followed by the incubation with HRP-conjugated secondary antibodies at a dilution of 1:10,000 [anti-mouse (Abcam, ab6789), anti-rabbit (Abcam, ab6721) and anti-goat (Abcam, ab6885)] for 2 h at room temperature. The membranes were washed in PBS plus 0.1% Tween 20, and protein detection was performed using enhancing chemiluminescence reagents. Glyceraldehyde-3-phosphate dehydrogenase (GAPDH) was used as a loading control.

### Data analysis

Images were captured on a Nikon Eclipse Ti multichannel confocal laser scanning system (Nikon, Japan). Z-series images were acquired at a nominal thickness of 1 μm (1024 × 1024 pixels). Image J software (National Institute of Health, Bethesda, MD, USA) was used to convert images from RGB to 8-bit binary; particles were then analyzed to obtain the percentage area of immunoreactivity [21]. For human samples, slides were coded, and immunohistochemistry images were quantified blindly. Statistical analysis was performed by Student *t-test* for a two-group comparison. For the correlation of markers expression with the clinicopathological parameters, the Cox regression test for survival analysis, the Chi-Square test, and multivariate correlation analyses were used. Pearson Correlation was performed to analyze the relationship between the overall expression of immunosuppressive with cholinergic markers. A chemiluminescent signal was captured for western blot using the FluorChem FC2 System (Alpha Innotech, USA). The expression level of each protein was quantified using ImageJ software (National Institute of Health, Bethesda, MD, USA). For apoptosis assay, BD FACs Canto II and FACS Diva software (BD Biosciences, Australia) was used to aid in analysis. *Two-way ANOVA* was used for multiple group comparison. Microsoft Excel, SPSS, and Prism (Graph Pad Software, La Jolla, CA, USA) were used to aid the statistical analysis, and *p* < 0.05 was considered significant.

## Results

### Expression of immunosuppressive markers in CRC tissues

#### Enhanced expression of PD-L1 but not PD-L2 associated with advanced stages of CRC

**Fig. 1:**
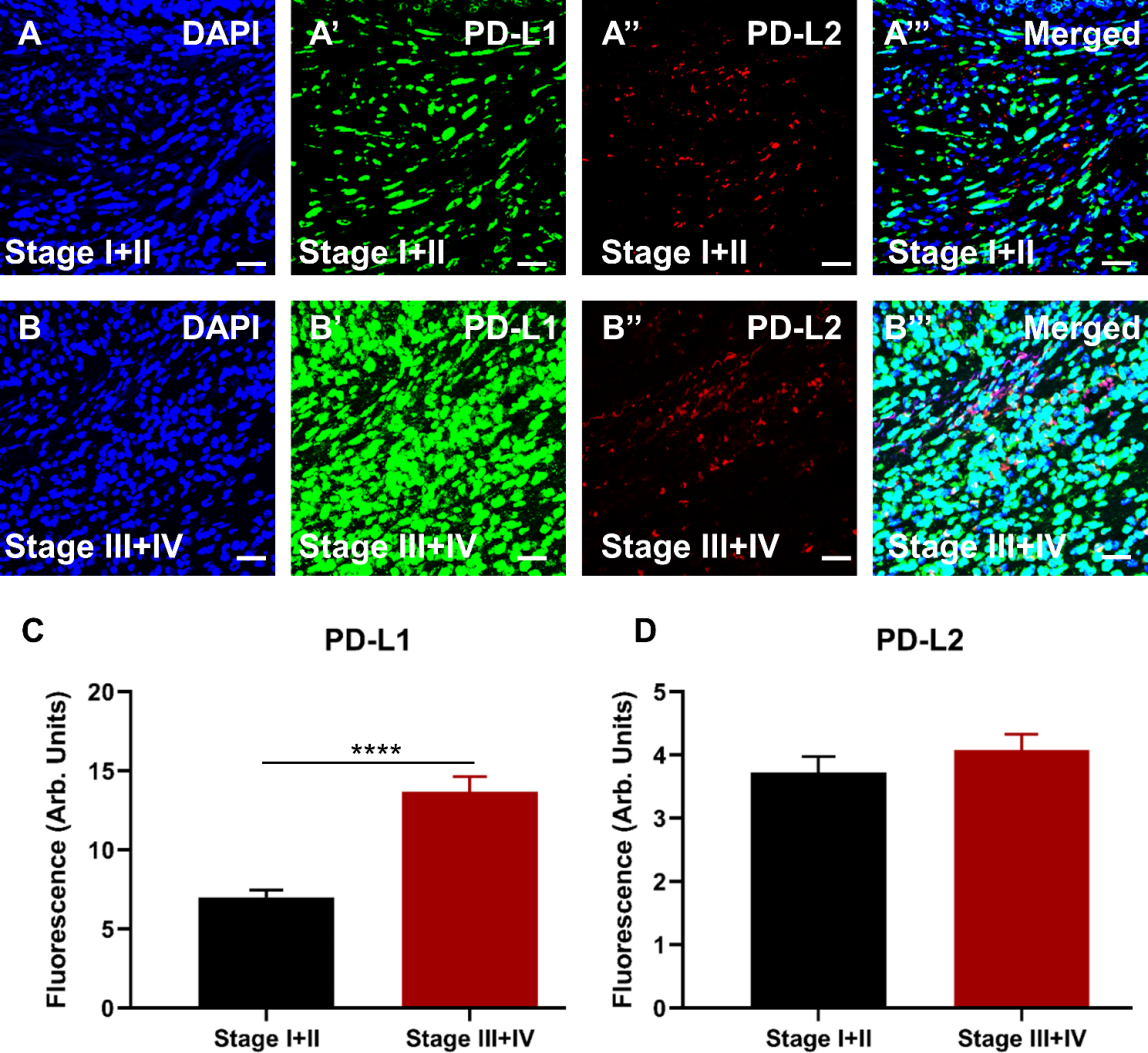
Expression of immunosuppressive markers PD-L1 and PD-L2 in human tissues from colorectal cancer (CRC) patients diagnosed with early stages, I + II (**A-A’’’**) and advanced stages, III + IV (**B-B’’’**). Tissues were labeled with the nuclei marker DAPI (blue; **A** and **B**), PD-L1 (green; **A’** and **B’**), PD-L2 (red; **A’’** and **B’’**), and all markers merged (**A’’’** and **B’’’**). The scale bar represents 50 μm. Bar graphs displaying the mean fluorescence of PD-L1 (**C**) and PD-L2 (**D**) in tissues from patients with early and advanced stages of CRC. Data presented as mean ± standard error of the mean (SEM), early stages I + II n = 49 and advanced stages III + IV n = 42. Student *t-*test, ****p < 0.0001

Colorectal cancer tissues were immunolabelled with antibodies for immunosuppressive markers, as described in the Methods section. DAPI expression is shown in Fig. 1A**&B**. Advanced stages of CRC abundantly expressed PD-L1 compared to early stages (I + II) (Fig. 1A**’-B’, C; ∆**: 6.67 ± 1.06, *p* < 0.0001). On the other hand, PD-L2 expression was not associated with CRC stages (Fig. 1A**’’-B”, D; ∆**: 0.35 ± 0.36, p = 0.3257). All markers merged are shown in Fig. 1A**’’’** and **B’’’**.

#### Clinicopathological and demographic parameters of CRC patients and their relevance to the expression of immunosuppressive markers

The average patient’s age was 65 years ranging from 33 to 91, and 51 male and 40 female patients were in the cohort. Among these patients, 49 were diagnosed with early clinical stages I + II and 42 with advanced stages III + IV of CRC.

The Chi-Square test was used to determine the correlation between the expression of immunosuppressive markers and clinicopathological parameters. PD-L1 expression was correlated with gender, age, and stage (Table 1). Out of 91 patients, 38 (41.8%) patients expressed low levels of PD-L1, and 53 (58.2%) expressed high levels. A significant difference was observed between the expression of PD-L1 and stages of CRC. In the early stages, 29 (31.9%) patients were noted to express low levels of PD-L1, and 20 (22.0%) expressed high levels of PD-L1, compared to advanced stages, where only 9 (9.9%) expressed low levels of PD-L1, and 33 (36.3%) expressed high levels of PD-L1. These findings suggest that high levels of PD-L1 are associated with advanced stages III + IV of CRC compared to early stages.

**Table 1:** Correlation of clinicopathological and demographic parameters of CRC patients with PD-L1 and PD-L2 expression

Parameters	No. of cases	Percentage (%)	PD-L1 expression Low High			*P* values
**Total**	91	100	38 (41.8%)		53 (58.2%)	
**Gender** Male Female	51 40	56.0 44.0	21 (23.1%) 17 (18.7%)		30 (30.0%) 23 (25.3%)	0.534
**Age** <65 >65	41 50	45.1 54.9	17 (18.7%) 21 (23.1%)		24 (26.0%) 29 (31.9%)	0.565
**Stage** I + II III + IV	49 42	53.8 46.2	29 (31.9%) 9 (9.9%)		20 (22.0%) 33 (36.3%)	0.0001
**Parameters**	**No. of cases**	**Percentage (%)**	**PD-L2 expression** **Low High**			***P*** **values**
**Total**	91	100	69 (75.8%)	22 (24.2%)		
**Gender** Male Female	51 40	56.0 44.0	39 (42.9%) 30 (33.0%)	12 (13.2%) 10 (11.0%)		0.531
**Age** <65 >65	41 50	45.1 54.9	31 (34.1%) 38 (41.8%)	10 (11.0%) 12 (13.2%)		0.579
**Stage** I + II III + IV	49 42	53.8 46.2	37 (40.7%) 32 (35.9%)	12 (13.2%) 10 (11.0%)		0.569

*P* values are based on the frequency of PD-L1 and PD-L2 expression within each parameter

There was no statistical difference observed in PD-L1 expression and patients’ gender, as 21 (23.1%) males and 17 (18.7%) females expressed low levels of PD-L1, whereas 30 (33.0%) males and 23 (25.3%) females expressed high levels. In addition, patients were divided into two age groups, under 65 (< 65) and over 65 (> 65) years old. Seventeen (18.7%) < 65 and 21 (23.1%) > 65 patients expressed low levels of PD-L1, 24 (26.0%) < 65, and 29 (31.9%) > 65 patients expressed high levels. Nevertheless, there was no significant correlation between the level of PD-L1 expression and patients’ age. On the other hand, 75.8% of patients expressed low levels of PD-L2 and 24.2% expressed high levels. Regarding patients’ age, gender, and stage, no significant differences were observed between the level of PD-L2 expression and these parameters (Table 1).

**Fig. 2:**
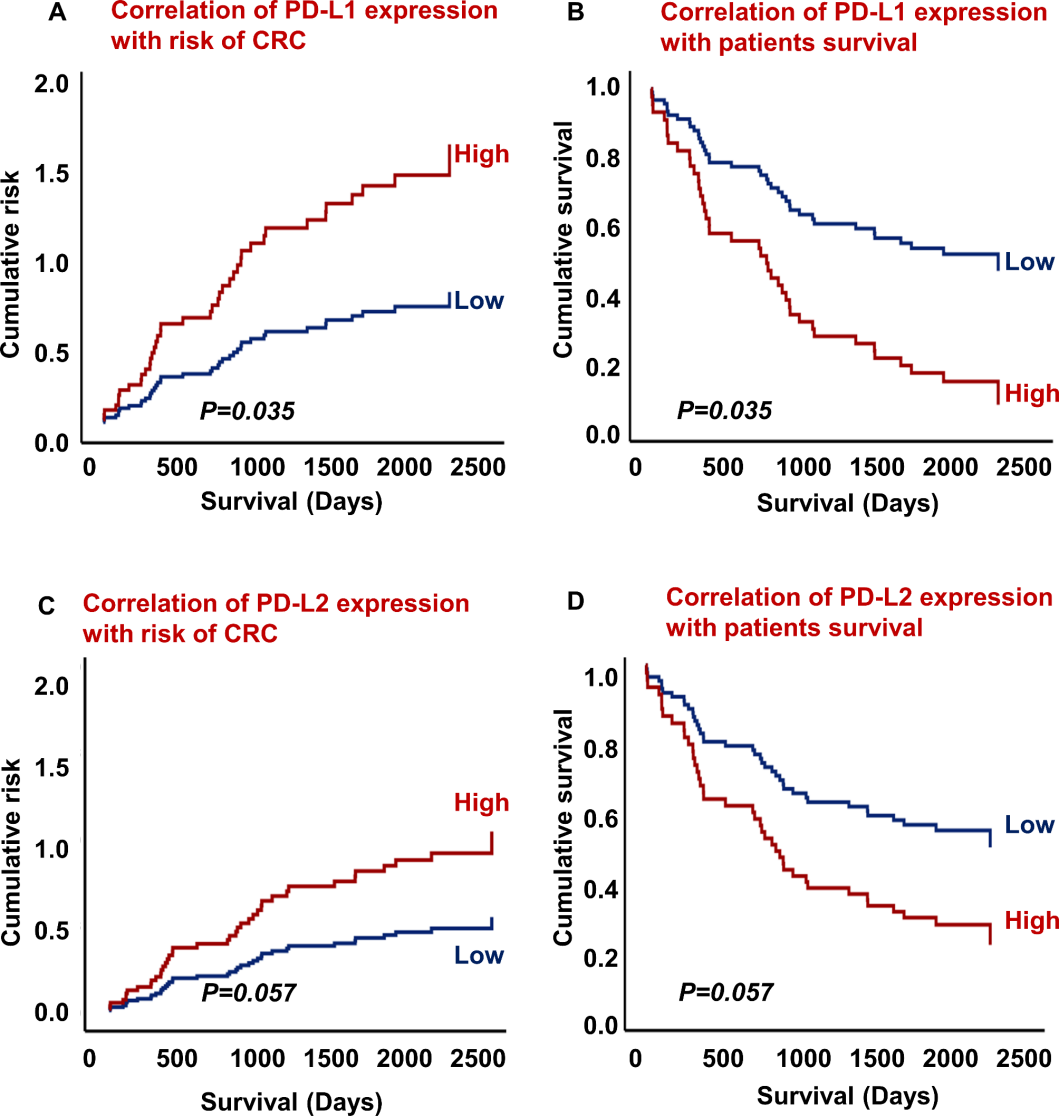
Correlation of PD-L1 expression with risk of CRC (**A**). PD-L1 expression association with survival outcomes (**B**). Correlation of PD-L2 expression with risk of CRC (**C**). PD-L2 expression association with survival outcomes (**D**). Early stages, I + II n = 49, and advanced stages, III + IV n = 42

Additionally, PD-L1 and PD-L2 expression was correlated with the risk of CRC and patients’ survival outcomes. This correlation was analyzed by hazard ratio (HR) and corresponding 95% confidence interval (CI) using Cox regression survival analysis. The results of this analysis demonstrated a significant correlation between high expression of PD-L1 and a high risk of CRC and poor survival outcomes (Fig. 2A, B, HR = 0.472, 95% CI = 0.236–0.949; *p =* 0.035). Furthermore, PD-L2 expression was not associated with CRC risk and poor patient survival outcomes (Fig. 2C, D, HR = 0.525, 95% CI = 0.271–1.021, *p =* 0.057).

### Expression of cholinergic markers in patients diagnosed with stages I-IV of CRC

**Fig. 3:**
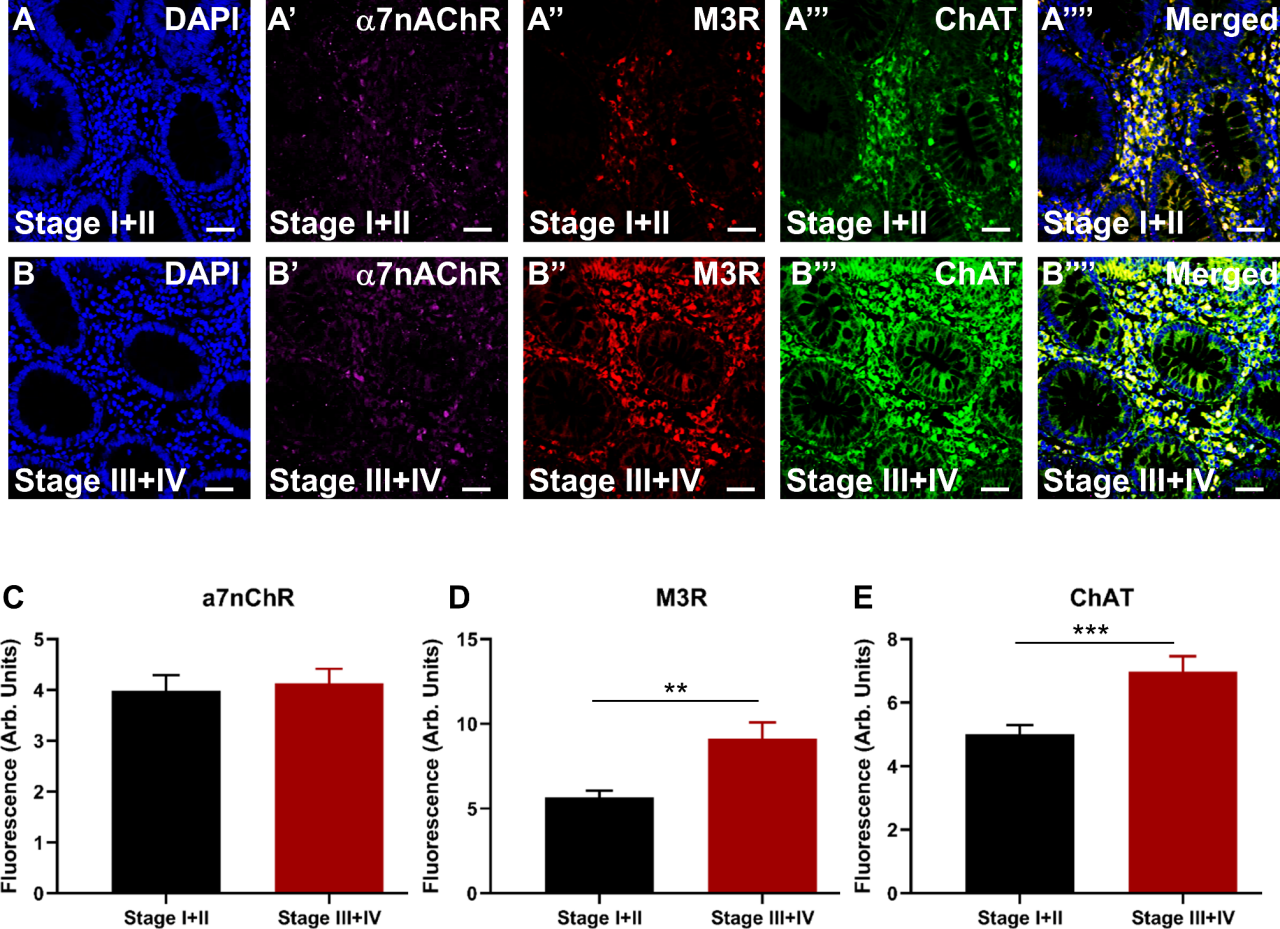
Expression of cholinergic markers, α7nAChR, M3R, and ChAT in tissues from patients diagnosed with early stages, I + II (**A-A’’’’**) and advanced stages, III + IV (**B-B’’’’**). Tissues were labeled with the nuclei marker DAPI (blue; **A** and **B**), a7nAChR (magenta; **A’** and **B’**), M3R (red; **A’’** and **B’’**), ChAT (green; **A’’’** and **B’’’**) and all markers merged (**A’’’’** and **B’’’’**). The scale bar represents 50 μm. Bar graphs displaying the mean fluorescence (arb. units) expression of α7nAChR (**C**), M3R (**D**), and ChAT (**E**) in patients with early stages, I + II, and advanced stages of CRC. Data presented as mean ± SEM, early stages, I + II, n = 49, and advanced stages, III + IV, n = 42. Student *t*-test, **p < 0.01, ***p < 0.001

The expression of cholinergic markers was evaluated in specimens obtained from patients diagnosed with early (I + II) and advanced (III + IV) CRC stages. DAPI expression is shown in Fig. 3A and **B**. No significant difference was observed in α7nAChR expression between early and advanced stages of CRC (Fig. 3A**’-B’, C; ∆**: 0.15 ± 0.45, *p =* 0.7264). Conversely, M3R expression was significantly increased at advanced stages, III + IV, compared to early stages, I + II (Fig. 3A**’’-B”, D; ∆**: 3.45 ± 1.05, *p =* 0.0015). Similarly, results also showed overexpression of ChAT at advanced stages, III + IV, compared to early stages, I + II (Fig. 3A**’’’-B’”, E; ∆**: 1.97 ± 0.56, *p =* 0.0006). All markers merged are shown in Fig. 3A**’’’’** and **3B’’’’**.

#### Correlation between cholinergic markers and clinicopathological and demographic parameters of CRC patients

The Chi-square test was utilized to determine the association between the expression of cholinergic markers and demographic and clinicopathological parameters of the patients diagnosed with CRC. The expression of α7nAChR was associated with patients’ age, gender, and stage of CRC. The results show that 49 (53.8%) patients expressed low levels of α7nAChR, while 42 (46.2%) expressed high levels. No association was noted between α7nAChR and patients’ age, gender, and stage (Table 2).

**Table 2:** Correlation of clinicopathological and demographic parameters of CRC patients with cholinergic markers

Parameters	No. of cases	Percentage (%)	α7nAChR expression Low High		*P* values
**Total**	91	100	49 (53.8%)	42 (46.2%)	
**Gender** Male Female	51 40	56.0 44.0	30 (33.0%) 19 (20.9%)	21 (23.1%) 21 (23.1%)	0.194
**Age** <65 >65	41 50	45.1 54.9	24 (26.4%) 25 (27.5%)	17 (18.7%) 25 (27.5%)	0.274
**Stage** I + II III + IV	14 33	15.4 36.3	30 (33.0%) 19 (20.9%)	19 (20.9%) 23 (25.3%)	0.094
**Parameters**	**No. of cases**	**Percentage (%)**	**M3R expression** **Low High**		***P*** **values**
**Total**	91	100	58 (63.7%)	33 (36.3%)	
**Gender** Male Female	51 40	56.0 44.0	31 (34.1%) 27 (29.7%)	20 (22.0%) 13 (14.3%)	0.508
**Age** <65 >65	41 50	45.1 54.9	22 (24.2%) 36 (39.6%)	19 (20.9%) 14 (15.4%)	0.070
**Stage** I + II III + IV	49 42	53.8 46.2	38 (41.8%) 11 (12.1%)	11 (12.1%) 31 (34.1%)	0.017
**Parameters**	**No. of cases**	**Percentage (%)**	**ChAT expression** **Low High**		***P*** **values**
**Total**	91	100	32 (35.2%)	59 (64.8%)	
**Gender** Male Female	51 40	56.0 44.0	30 (33.0%) 19 (20.9%)	21 (23.1%) 21 (23.1%)	0.194
**Age** <65 >65	41 50	45.1 54.9	24 (26.4%) 25 (27.5%)	17 (18.7%) 25 (27.5%)	0.274
**Stage** I + II III + IV	49 42	53.8 46.0	30 (33.0%) 14 (15.4%)	19 (20.9%) 28 (30.6%)	0.049

*P* values are based on the frequency of cholinergic markers within each parameter

High levels of M3R expression are associated with the stage of CRC but not with patients’ age and gender (Table 2). Early stages of CRC, I + II, mostly expressed low levels of M3R as 41.8% of patients expressed low levels, while 12.1% expressed high levels. Whereas in advanced stages, III + IV, 34.1% expressed high levels of M3R, and 12.1% expressed low levels, suggesting that high levels of M3R expression might hold a prognostic value for an advanced stage of CRC.

Furthermore, out of 91 patients, 32 (35.2%) expressed low levels of ChAT, and 59 (64.8%) expressed high levels. A high level of ChAT expression was not associated with the patient’s age and gender but with CRC stages (Table 2). In the early stages, I + II, 33% of the patients expressed low levels, and 20.9% expressed high levels, whereas, in advanced stages, III + IV, 15.4% of the patients expressed low levels, and 30.6% expressed high levels of ChAT.

**Fig. 4:**
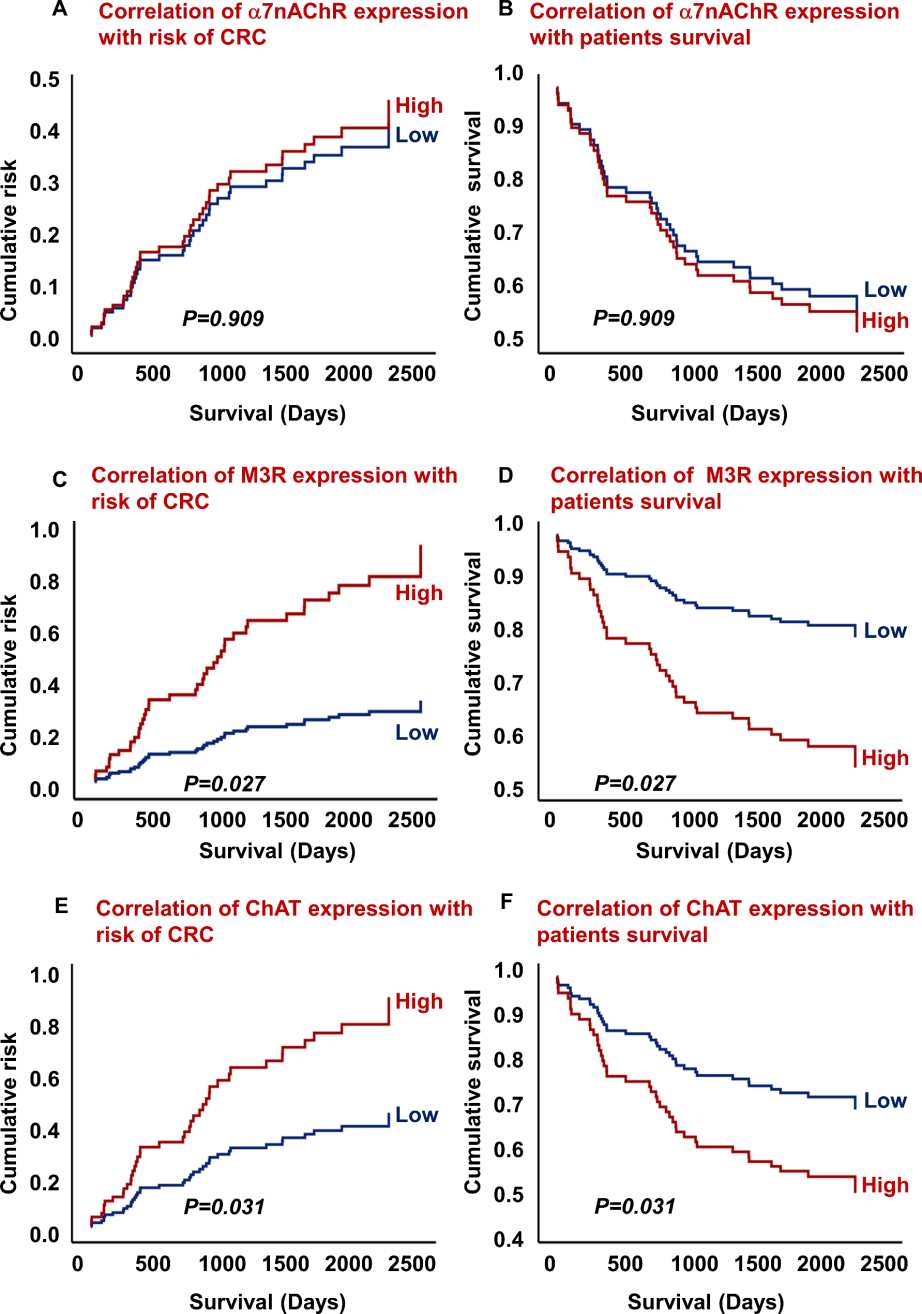
Correlation of α7nAChR expression with risk of CRC (**A**) and survival outcomes (**B**). Correlation of M3R expression with risk of CRC (**C**) and survival outcomes (**D**). Association of ChAT expression with risk of CRC (**E**) and survival outcomes (**F**). Early stages, I + II, n = 49 and advanced stages, III + IV, n = 42

In addition, the correlation of α7nAChR, M3R, and ChAT expression with CRC risk and patients’ survival outcomes was analyzed. There was no correlation between the expression of α7nAChR, risk of CRC, and patients’ survival outcomes (Fig. 4A, B, HR = 0.909, 95% CI = 0.479–1.727; *p =* 0.909). A significant correlation was observed between the high expression of M3R and a high risk of CRC and poor survival outcomes (Fig. 4C, D, HR = 0.346, 95% CI = 0.135–0.884; *p =* 0.027). Furthermore, high levels of ChAT expression were also associated with a higher risk of CRC and poor patient survival outcomes (Fig. 4E, F, HR = 0.501, 95% CI = 0.267–0.940; *p =* 0.031).

### Correlation between immunosuppressive and cholinergic markers

We further evaluated the overall correlation between immunosuppressive markers’ expression with cholinergic markers (Table 3). Overall, the expression of PD-L1 was strongly correlated with M3R expression, moderately associated with PD-L2 and ChAT, while weakly correlated with α7nChR expression. In contrast, PD-L2 overall expression was strongly associated with α7nAChR, moderately correlated with PD-L1 and ChAT, but not with M3R expression. Moreover, α7nAChR expression was strongly associated with PD-L2 and ChAT while weakly correlated with PD-L1 and M3R. Furthermore, M3R was strongly correlated with PD-L1 and ChAT, whereas weakly associated with α7nAChR. Overall, the expression of ChAT was strongly correlated with cholinergic receptors and moderately associated with immunosuppressive markers. These findings suggest that there might be crosstalk between immunosuppressive markers with cholinergic markers.

**Table 3:** Correlation of immunosuppressive markers with cholinergic markers in CRC patients

		PD-L1	PD-L2	M3R	α7nAChR	ChAT
**PD-L1**	Pearson Correlation	1	0.351**	0.562**	0.236*	0.324**
	Sig. (2-tailed)		0.001	0.000	0.025	0.002
	N	91	91	91	91	91
**PD-L2**	Pearson Correlation	**0.351** ^**^	1	**0.153**	**0.561** ^**^	**0.370** ^**^
	Sig. (2-tailed)	0.001		0.147	0.000	0.000
	N	91	91	91	91	91
**M3R**	Pearson Correlation	**0.562** ^**^	0.153	1	**0.298** ^**^	**0.571** ^**^
	Sig. (2-tailed)	0.000	0.147		0.004	0.000
	N	91	91	91	91	91
**α7nAChR**	Pearson Correlation	**0.236** ^*^	**0.561** ^**^	**0.298** ^**^	1	**0.679** ^**^
	Sig. (2-tailed)	0.025	0.000	0.004		0.000
	N	91	91	91	91	91
**ChAT**	Pearson Correlation	**0.324** ^**^	**0.370** ^**^	**0.571** ^**^	**0.679** ^**^	1
	Sig. (2-tailed)	0.002	0.000	0.000	0.000	
	N	91	91	91	91	91
* Correlation is significant at the 0.05 level (2-tailed).						
** Correlation is significant at the 0.01 level (2-tailed).						

Strong correlation, ± 0.50 and ± 1; Moderate correlation, ± 0.30 and ± 0.49; Weak correlation, ± 0.29

### Effects of blocking muscarinic receptors on proliferation, migration, apoptosis, and choline production in normal intestinal epithelial and human colon cancer cells

**Fig. 5:**
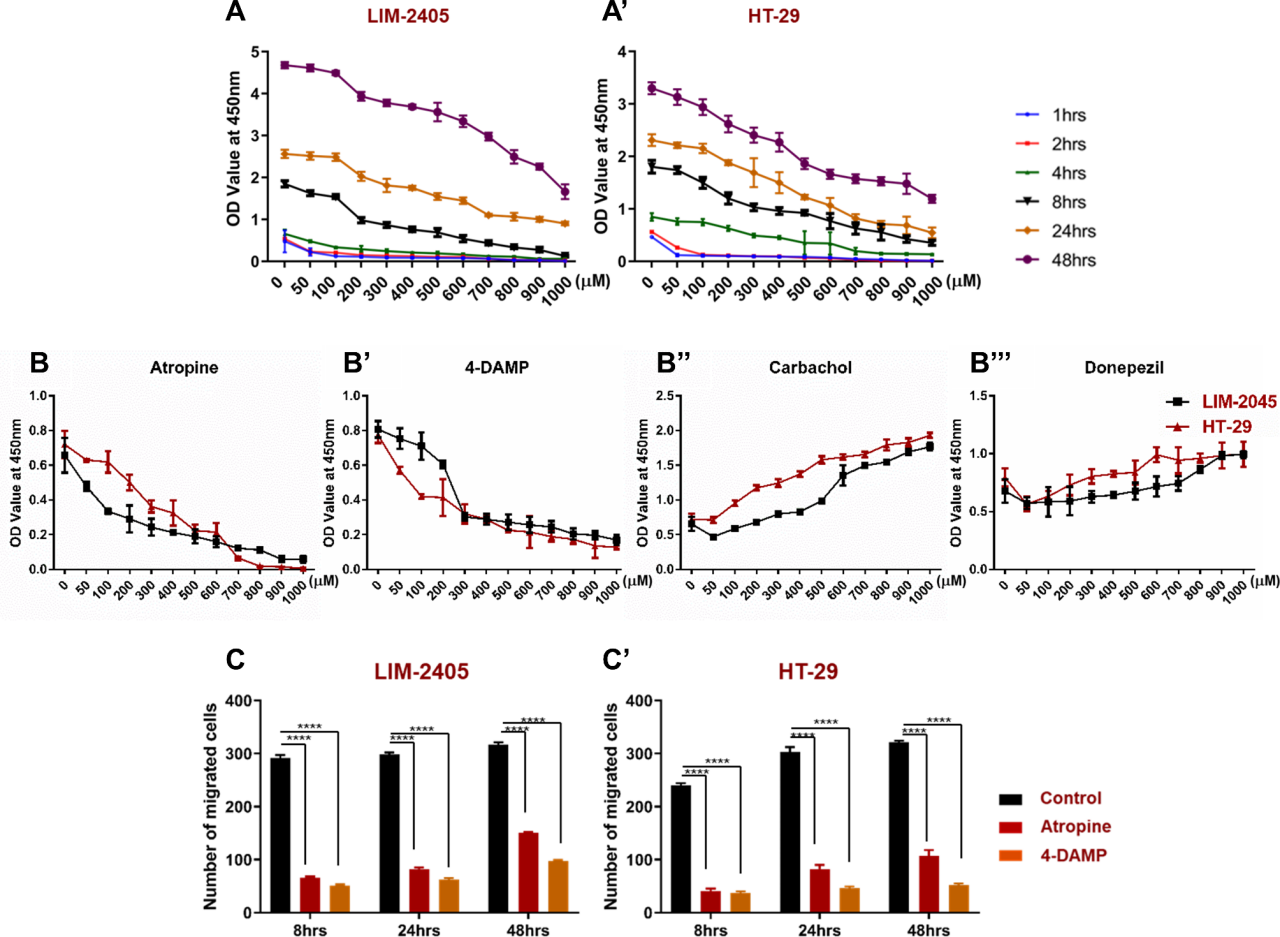
Cell proliferation dose-response curve for LIM-2405 (**A**), and HT-29 (**A’**) cells treated with atropine for 1–48 hours (hrs). The proliferation of LIM-2405, and HT-29 cell lines treated with atropine (**B**), 1,1-dimethyl-4 diphenylacetoxypiperidinium iodide (4-DAMP) (**B’**), carbachol (**B”**) and donepezil (**B”’**) for 8 h. Boyden chamber was used to determine the effect of atropine and 4-DAMP on the migration of LIM-2405 (**C**) and HT-29 (**C’**) cells for 8, 24, and 48 h. Values in **A-B”’** are mean ± standard error of the mean (SEM) from at least three independent experiments. Values in **C-C’** are presented as mean ± SEM from at least two independent experiments in duplicates. *Two-way ANOVA* followed by Tukey’s multiple comparisons test was used, and the significance value is marked with asterisks, ****p < 0.0001

Cellular proliferation is an essential step in the development and progression of cancer. Non-neuronal ACh plays a crucial role in colon cancer cell proliferation [22]. To determine the effect of blocking muscarinic receptors, cells were treated with various concentrations of atropine and at different time points. The effect of atropine on cellular proliferation of two human colon cancer cell lines (LIM-2405 and HT-29) was assessed using WST-1 assay. Three independent experiments were performed in triplicate wells. Atropine significantly decreased cell proliferation of all cells at 1–4 h compared to 8–48 h (Fig. 5A-A**’**). Though there was a trend of reduced proliferation at 8–48 h, atropine decreased proliferation in a dose-dependent manner. In all subsequent experiments, cells were treated with 100 μm of atropine for 8 h as changes were noticeable at this concentration and time point with high cell viability, reducing the auto-fluorescence staining from dead cells.

LIM-2405 and HT-29 cells were incubated with various concentrations of atropine and 4-DAMP for 8 hrs. In addition, cells were incubated with various concentrations of carbachol that act as ACh receptor agonist and donepezil, inhibiting ACh breakdown. Figure 5B-B**"’** shows the proliferation of two human colon cancer cells treated with atropine, 4-DAMP, carbachol, and donepezil. Atropine inhibited the proliferation of all cells in a dose-dependent manner; however, LIM-2405 cells were more sensitive to atropine than HT-29 cells (Fig. 5B). Similarly, a 4-DAMP suppressed proliferation of all cells, but HT-29 cells were more sensitive to the 4-DAMP effect than LIM-2405 cells (Fig. 5B**’**). To determine if ACh agonists can reverse the effect of atropine and 4-DAMP, cells were incubated with carbachol and donepezil. The results show that carbachol and donepezil increased cell proliferation in a dose-dependent fashion (Fig. 5B**”-B"’**).

Cell migration is essential in cancer progression as it is the main hallmark of cancer metastasis. The effects of atropine and 4-DAMP on LIM-2405 and HT-29 ability to migrate was evaluated using the Boyden chamber. Cells were incubated with 100 µM of atropine and 4-DAMP for 8, 24, and 48 h (Fig. 5C-C**’**). Both atropine and 4-DAMP decreased the migration of LIM-2405 and HT-29 cells compared to controls at all time points (Fig. 5C-C**’**).

**Fig. 6:**
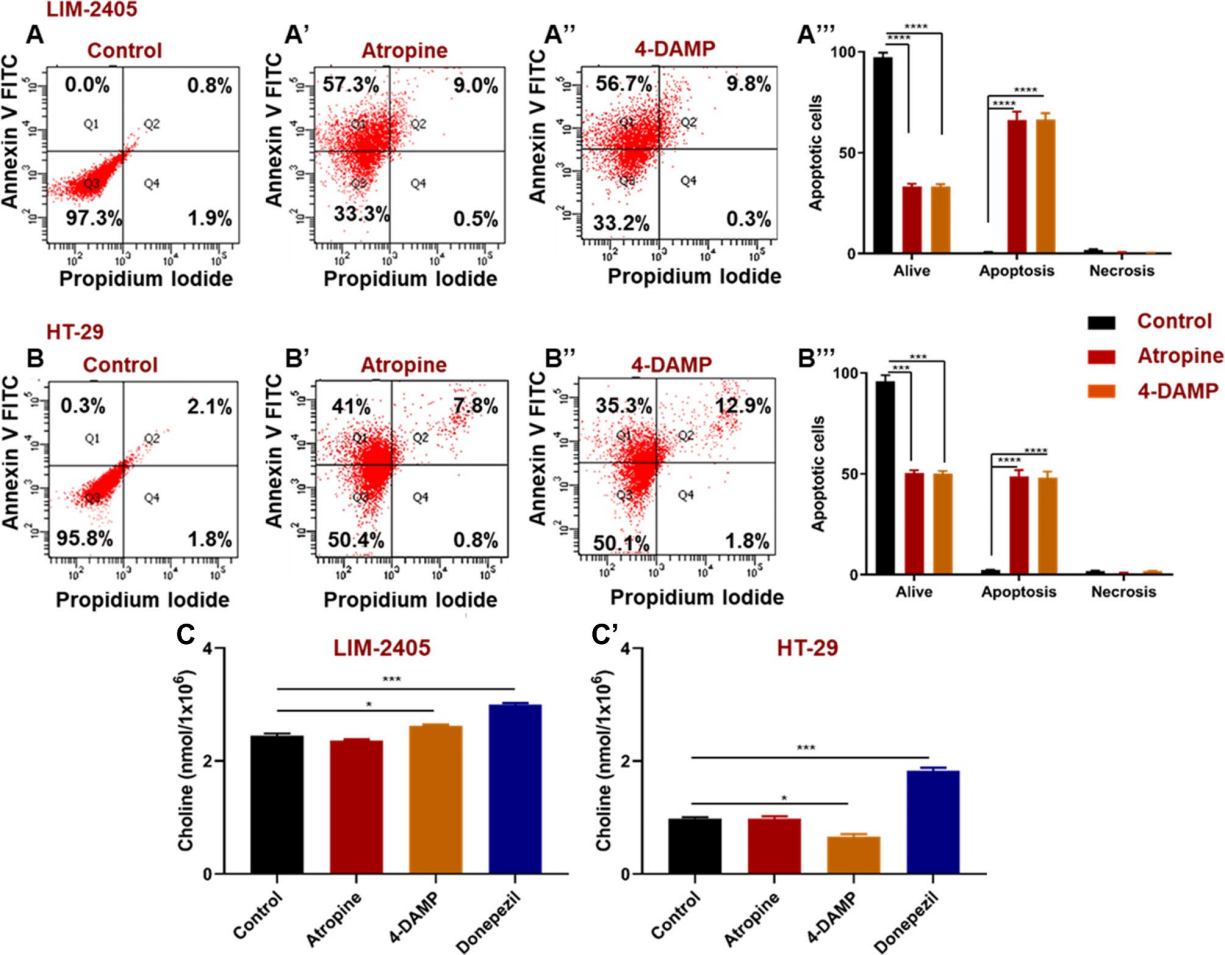
Annexin V-FITC/propidium iodide (PI) staining of human colon cancer cell lines incubated with atropine (**A’**, **B’**) and 4-DAMP (**A”**, **B’’**). (**A**) LIM-2405 controls, (**A’**) LIM-2405 treated with atropine, (**A’’**) LIM-2405 treated with 4-DAMP, and the percentage of cells treated with control, atropine, and 4-DAMP for LIM-2405 (**A”’**), (**B**) HT-29 controls, (**B’**) HT-29 treated with atropine, (**B’’**) HT-29 treated with 4-DAMP and the percentage of cells treated with control, atropine and 4-DAMP for HT-29 (**B”’**). The amount of choline was measured in LIM-2056 (**C’**) and HT-29 (**C’**) cells. Values in **A-B”’** are mean ± standard error of the mean (SEM) from at least three independent experiments performed in triplicate wells. Values in **C-C’** presented as mean ± SEM from at least two independent experiments in duplicates. *Two-way ANOVA* followed by Tukey’s multiple comparisons test was used, and the significance value is marked with asterisks, *p < 0.05, ***p < 0.001, ****p < 0.0001

Acetylcholine receptors, especially muscarinic receptors, play a significant role in regulating cell apoptosis [22]. To determine whether atropine and 4-DAMP induce apoptosis or necrosis in LIM-2405 and HT-29 cancer cell lines, cells were incubated with Annexin V and propidium iodide (PI). Non-apoptotic cells are Annexin V and PI negative, while apoptotic cells are Annexin V positive and PI negative. Necrotic cells are PI-positive and Annexin V-negative. Blockade of all muscarinic receptors with atropine and the selective block of M3R with 4-DAMP induced apoptosis, but not necrosis, in both cancer cell lines (Fig. 6A-B**"’**).

Cancer cells synthesize their own ACh. To determine whether LIM-2405 and HT-29 cells could synthesize ACh, the amount of choline, a precursor for ACh synthesis, was measured in cell lysate (1 × 10^6^ cells). A choline/acetylcholine assay kit, which is rapid, sensitive, and accurate, was used to measure choline in the cell lysate. There were no significant differences between cells treated with atropine compared to controls (Fig. 6C-C**’**). However, 4-DAMP significantly increased choline production in LIM-2405 cells (Fig. 6C), while HT-29 cells showed decreased choline compared to the control (Fig. 6C**’**). In addition, cells were treated with acetylcholinesterase inhibitor donepezil to determine its effects on LIM-2405 and HT-29 cells, showing that donepezil significantly augmented choline production in both cell lines (Fig. 6C-C**’**).

### Effect of atropine and 4-DAMP on the expression of immunosuppressive and cholinergic markers

**Fig. 7:**
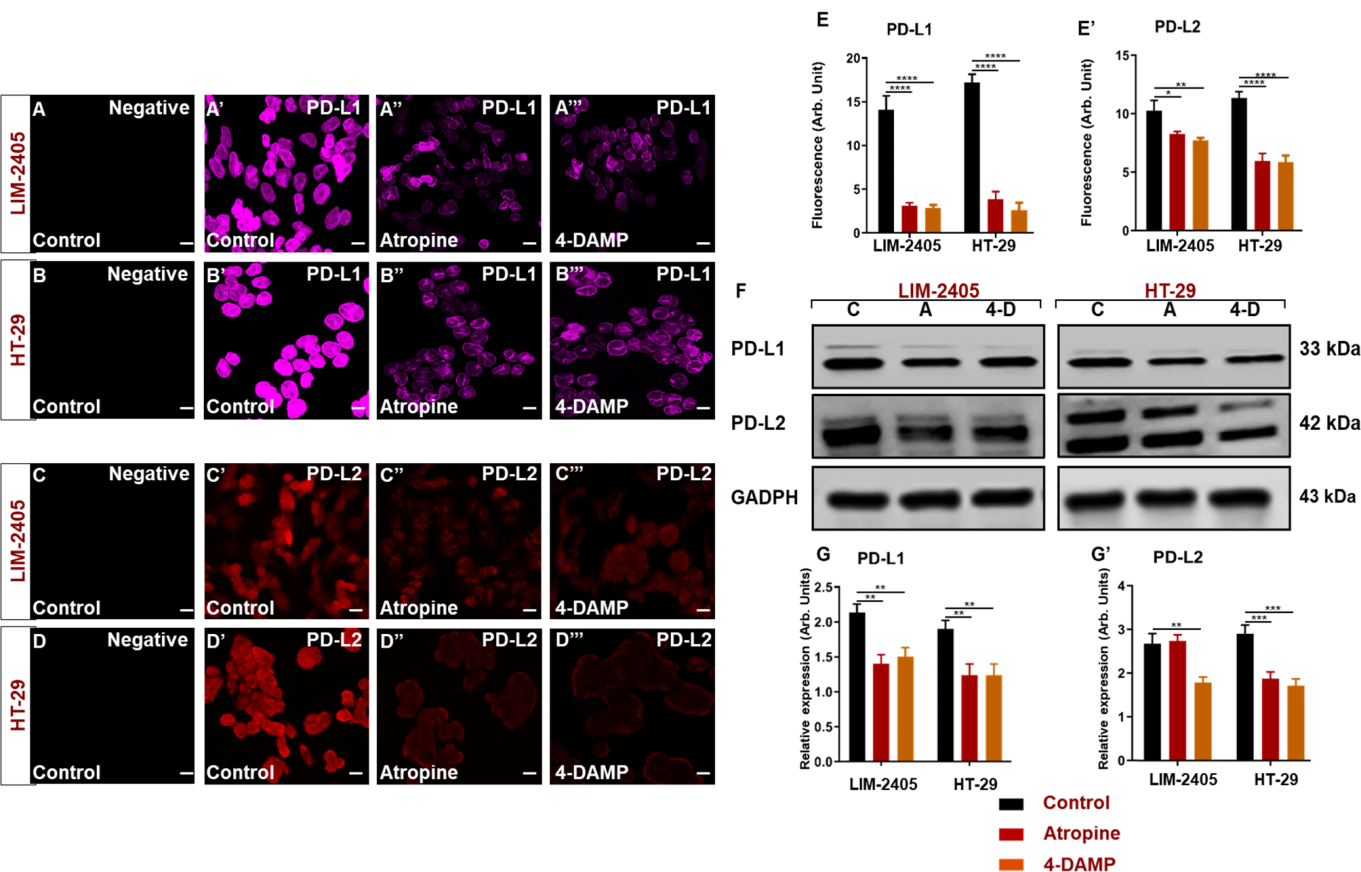
Negative control labeling of LIM-2405 cells is presented in (**A**), control (**A’**), atropine (**A’’**), and 4-DAMP (**A’’’**). HT-29 negative control is displayed in (**B**), control (**B’**), atropine (**B’’**), and 4-DAMP (**B’’’**). Negative control labeling of LIM-2405 cells is presented in (**C**), control (**C’**), atropine (**C’’**), and 4-DAMP (**C’’’**). HT-29 negative control is displayed in (**D**), control (**D’**), atropine (**D’’**), and 4-DAMP (**D’’’**). The scale bar represents 50 μm. Bar graphs displaying the mean fluorescence of PD-L1 (**E**), PD-L2 (**E’**); western blot bands for LIM-2405, and HT-29 ran on the same blot with a well separating each cell line, labeled with PD-L1 and PD-L2 are shown in (**F**), and western blot expression intensity (**G-G’**). C: control, A: atropine, 4-D: 4-DAMP. Data presented as mean ± SEM. Two-way ANOVA, *p < 0.05, **p < 0.01, ***p < 0.001, ****p < 0.0001

The muscarinic receptor antagonist atropine has been reported to inhibit cancer cell growth both in vitro and in vivo [23]. However, no studies have shown the effects of muscarinic acetylcholine receptor (mAChR) blocking on the expression of immunosuppressive markers, PD-L1 and PD-L2. LIM-2405 and HT-29 were used to determine the effect of atropine on the expression of these markers. The PD-L1 and PD-L2 expression levels were evaluated by immunofluorescence and western blot analyses. Cells were pre-treated with 0 and 100 µM atropine and 4-DAMP for 8 hr followed by incubation with the antibody diluent with or without primary antibodies, followed by incubation with secondary antibodies. No labeling of cells was observed without primary antibodies (Fig. 7A-D), indicating that the positive labeling is specific to the primary antibodies of interest. Atropine significantly decreased PD-L1 expression in LIM-2405 (Fig. 7A**”, E-G**) and HT-29 (Fig. 7B**”, E-G**) cells compared to controls (Fig. 7A**’-B’, E-G**). 4-DAMP decreased PD-L1 in LIM-2405 (Fig. 7A**’”, E-G**) and HT-29 (Fig. 7B**’”, E-G**) cells. Likewise, the effect of atropine and 4-DAMP on the expression of PD-L2 was evaluated (Fig. 7C-D**’”**). Atropine significantly decreased the expression of PD-L2 in LIM-2405 (Fig. 7C**”, E’-G’**) and HT-29 (Fig. 7D**”, E’-G’**) compared to controls (Fig. 7C**’-D’, E’-G’**). 4-DAMP decreased PD-L2 in LIM-2405 (Fig. 7C**’”, E’-G’**) and HT-29 (Fig. 7D**’”, E’-G’**) cells. Interestingly, LIM-2405 displayed one molecular band, while in HT-29 two molecular bands were observed. We speculate that the difference in molecular bands could be due to molecular differences between these two cells.

**Fig. 8:**
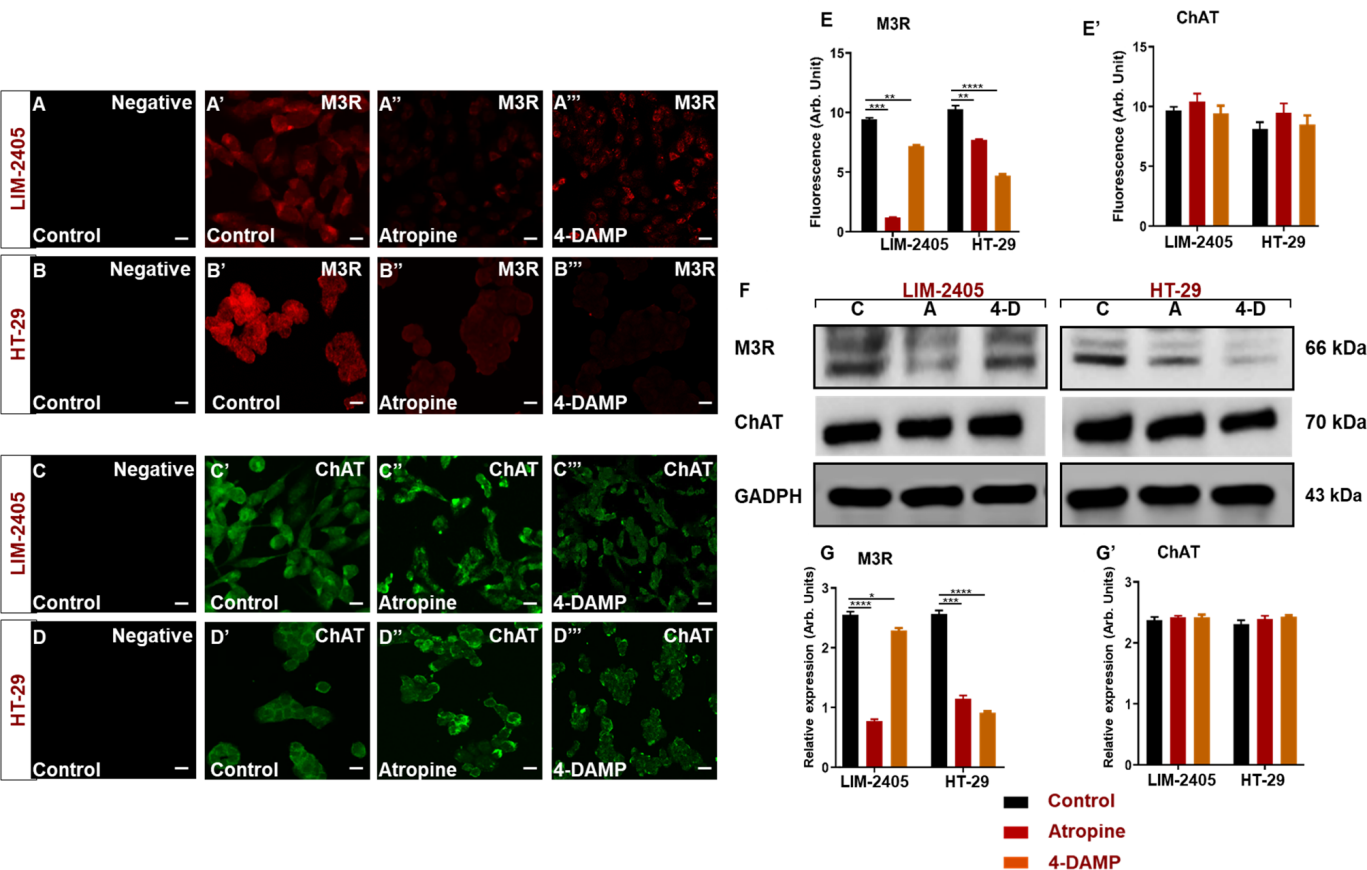
Negative control labeling of LIM-2405 cells is presented in (**A**), control (**A’**), atropine (**A’’**), and 4-DAMP (**A’’’**). HT-29 negative control is displayed in (**B**), control (**B’**), atropine (**B’’**), and 4-DAMP (**B’’’**). Negative control labeling of LIM-2405 cells is presented in (**C**), control (**C’**), atropine (**C’’**), and 4-DAMP (**C’’’**). HT-29 negative control is displayed in (**D**), control (**D’**), atropine (**D’’**), and 4-DAMP (**D’’’**). The scale bar represents 50 μm. Bar graphs displaying the mean fluorescence of M3R (**E**), ChAT (**E’**); western blot bands for LIM-2405, and HT-29 ran on the same blot with a well separating each cell line, labeled with M3R and ChAT are shown in (**F**) and western blot expression intensity is shown in (**G-G’**). C: control, A: atropine, 4-D: 4-DAMP. Data presented as mean ± SEM. Two-way ANOVA, *p < 0.05, **p < 0.01, ***p < 0.001, ****p < 0.0001

Muscarinic receptors, particularly M3R, play a significant role in the progression of CRC. The effect of atropine and 4-DAMP on M3R and ChAT expression was evaluated in human colon cancer cell lines, LIM-2405 and HT-29, by immunofluorescence and western blot analyses (Fig. 8A-G**’**). Atropine treatment significantly reduced M3R expression in all cell lines (Fig. 8A**”-B”, E-G**). Specific blocking of M3R with 4-DAMP significantly decreased M3R in LIM-2405 (Fig. 8A**’”, E-G**) and HT-29 (Fig. 8B**’”, E-G**) cells when compared to the control. Blocking all muscarinic receptors with atropine did not affect the expression of ChAT in human colon cancer cell lines treated with atropine (Fig. 8C**”, D”, E’-G’**). Similarly, specific blocking of M3R with 4-DAMP did not affect the expression of ChAT in colon cancer cell lines (Fig. 8C**’”, D’”, E’-G’**).

### Effect of atropine and 4-DAMP on EGFR and phosphorylation of STAT3 and ERK kinases

It is known that muscarinic receptors suppress cell apoptosis through the activation of phosphatidylinositol-3-OH (PI3) kinase and its downstream targets, protein kinase B (PKB)/AKT and MAPK/ERK [24, 25]. Furthermore, the activation of signaling pathways is essential in the development of CRC.

**Fig. 9:**
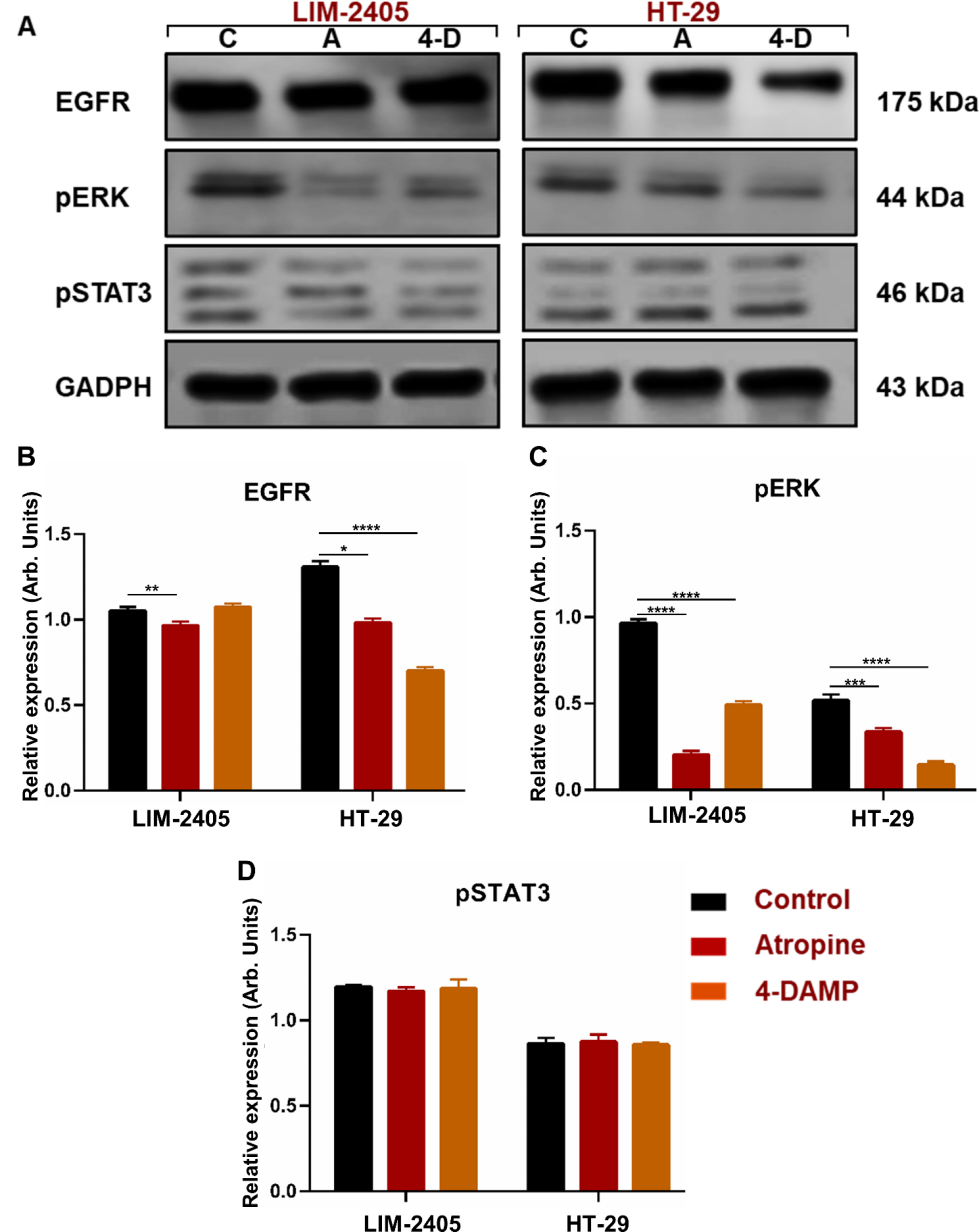
Western blot bands for LIM-2405, and HT-29 ran on the same blot with a well separating each cell line are shown in (**A**) (C: control, A: atropine, 4-D: 4-DAMP). The mean relative expression of EGRF (**B**), pERK (**C**), and pSTAT3 (**D**). Bar graphs are presented as mean ± SEM. Two-way ANOVA, *p < 0.05, **p < 0.01, ***p < 0.001, ****p < 0.0001

To gain insights into atropine and 4-DAMP mechanisms of action, immunoblotting of pSTAT3, pERK, and EGFR was performed. Atropine treatment significantly reduced EGFR expression in LIM-2405 and HT-29 cell lines (Fig. 9A, B). 4-DAMP treatment showed a trend towards increased EGFR expression in LIM-2405 cells, but not significant. However, in the HT-29 cell line, 4-DAMP attenuated EGFR expression compared to the control. Both atropine and 4-DAMP significantly decreased pERK expression in all cell lines (Fig. 9A, C). Atropine and 4-DAMP showed no effect on the phosphorylation of STAT3 in human colon cancer cell lines (Fig. 9A, D).

Overall, the data suggest that atropine exhibits its effect by inhibiting EGFR and pERK in human colon cancer cell lines. Similarly, blocking M3R with 4-DAMP exerts its effects via decreasing EGFR in HT-29 cells and inhibiting ERK phosphorylation in all cells. Hence, these findings suggest that atropine and 4-DAMP suppress the expression of immunosuppressive and cholinergic markers, cellular proliferation, migration, and induce apoptosis via EGFR/ ERK signaling pathways.

## Discussion

Cancer cells downregulate the hosts’ immune system by expressing PD-L1 and PD-L2, which interact with the PD-1 receptor on tumor-infiltrating lymphocytes [26]. PD-L1 and PD-L2 on the surface of cancer cells function as an immune resistance mechanism allowing cancer cells to go undetected, leading to their proliferation and growth. Our results demonstrate increased levels of PD-L1, M3R, and ChAT at advanced stages of CRC, which correlates with a high risk of CRC and poor survival outcomes independent of patients’ gender and age. On the contrary, PD-L2 expression was not associated with the stages of CRC. M3R and ChAT were also elevated at advanced stages, III + IV, compared to early stages, I + II. α7nAChR expression was not associated with stages, risk, and survival outcomes of CRC. In addition, in vitro findings show that immunosuppressive molecules, PD-L1 and PD-L2, were significantly elevated in human colon cancer cell lines. Treatment with atropine and a selective M3R blocker, 4-DAMP, decreased cell proliferation and migration as well as induced apoptosis. These effects involved suppressing PD-L1 and PD-L2 expression in cancer cells via inhibiting EGFR activation and phosphorylation of ERK protein kinases.

Overexpression of PD-L1 has been observed in many cancers, including CRC [27]. The increase in the expression of PD-L1 on cancer cells could be influenced by the immune microenvironment, thus allowing immune evasion [28]. As CRC progresses from stage I to stage IV, the expression of immunosuppressive molecules such as PD-L1 and PD-L2 would be expected to be significantly upregulated. Generally, it is conceivable to believe that patients with the advanced stage of CRC have an overall increase in the expression of immunosuppressive molecules to facilitate tumor evasion of the host’s immune system. In this study, PD-L1 expression was significantly enhanced at advanced stages, III + IV, compared to the early stages, I + II, of CRC. These findings are in line with studies demonstrating an association with cancer stages. For instance, high PD-L1 expression was associated with tumor node metastasis, poor prognosis, and shorter survival in CRC patients [29, 30]. Our findings reported a correlation between PD-L1 expression with poor prognosis and shorter survival in CRC patients, concurring with previous studies.

In contrast, some studies have demonstrated that the expression of PD-L1 on immune cells has a favorable prognostic factor in some cancers [31]. For example, PD-L1 expression associated with early stages, lower cancer grade, absence of vascular invasion, and lymph node metastasis significantly improved patient survival in mismatch repair-proficient microsatellite stable CRCs via enhanced CD8 + T cell infiltration [32]. These studies suggest that the prognostic value of PD-L1 expression could depend on the subset of CRCs and the presence of infiltrating immune cells. Furthermore, these contradictions in findings could be attributed to the expression of other markers, as we found a correlation between the expression of immunosuppressive and cholinergic markers in this study.

PD-L1 can be induced by signaling molecules such as nuclear factor-kappa B, mitogen-activated protein kinase, phosphoinositide 3-kinase, mammalian target of rapamycin, and Janus kinase/signal transducer and activator of transcription, providing a pathway for cancer cell evasion [33]. The present study demonstrates that atropine exhibits its effect by suppressing EGFR and pERK in LIM-2405 and HT-29. Similarly, blocking of M3R with 4-DAMP induced apoptosis, reduced cellular proliferation, migration, and the expression of immunosuppressive and cholinergic markers via inhibiting phosphorylation of ERK in LIM-2405 and via suppressing activation of EGFR and ERK phosphorylation in HT-29. Studies have shown that INF-γ partially induces most immunosuppressive markers of interest and oncogenic signaling pathways, including EGFR, ERK, and STAT3. The present study shows that Ach acting on M3R and PD-L1 acting on PD1 involve the same molecular mechanisms, that lead to cancer progression. Moreover, this study demonstrates that cholinergic blockers, and specifically M3R blocker, inhibit PD-L1 release, consistent with Kamiya et al. (2019) study Nature study. Therefore, activating these signaling pathways is essential in the development of CRC. STAT3 and ERK play a key role in cancer cell proliferation and migration. Hence, it is crucial to determine the effects of atropine and 4-DAMP on their expression.

The expression of PD-L2 in cancer is not well understood, as there are scarce studies identifying the role of PD-L2 in cancer progression. However, studies have reported that about 40% of cancer tissues from CRC patients overexpressed PD-L2 [34]. Studies demonstrated that PD-L2 exerts its function in immune tolerance by modulating and dampening the T-helper type 2 (Th2) response; however, the Th1 response is crucial for anti-cancer immunity [35, 36]. PD-L2 expression in esophageal squamous cell carcinoma is negatively associated with PD-1 positive tumor-infiltrating lymphocytes, suggesting a role in the escape mechanism of cancer cells from the host’s immune system [37]. PD-L2 expression is associated with poor survival in patients with esophageal cancer [38]. In renal cell carcinoma, PD-L2 expression was associated with shorter progression-free survival [39]. PD-L2 expression was independently associated with poor survival of CRC patients [40]. In the present study, PD-L2 expression was not associated with stage, risk of CRC, and patient survival outcome.

Similarly, several studies found no correlation between PD-L2 expression and survival outcomes, as noted in hepatocellular carcinoma, pancreatic and ovarian cancer patients [41–43]. In esophageal adenocarcinomas, PD-L2 expression is associated with smaller tumor size, early stages, and well-differentiated grade; however, PD-L2 is not associated with lymph node infiltration or metastasis patient survival [44]. This could be due to the small number of patients in these studies reporting PD-L2 expression. Furthermore, studies have shown that depending on the molecules present in the microenvironment, the expression of PD-L2 can be increased in immune and non-immune cells [45]. In the present study, PD-L2 was strongly associated with α7nAChR expression and moderately correlated with PD-L1 and ChAT but not with M3R. This suggests that PD-L2 might be influenced by ACh binding to α7nAChR but not M3R activation. Treating HCT116 human colon cancer cells with PD-L2 Fc fusion protein increased their invasion ability [46].

The role of ACh in cancer immunomodulation is not clear; however, treating spleen cultures with ACh enhances T cell proliferation, suggesting a possible role of ACh in activating an anti-cancer immune response [47, 48]. Many studies have reported that ACh and other constituents of cholinergic signaling, including ChAT and cholinergic receptors, are present in various non-neuronal tissues and many cancers [17, 23, 49, 50]. In addition, ACh plays an important role in cellular proliferation, migration, and apoptosis, which are essential for cancer development and progression. For instance, ChAT is upregulated in non-small cell lung carcinoma (NSCLC), while cholinesterase enzymes are downregulated, leading to increased ACh in cancer tissues [51, 52]. The data herein show that blocking muscarinic receptors decreased cellular proliferation and migration. This concurs with previous studies demonstrating the role of ACh in cellular proliferation and migration. Administration of a non-selective muscarinic receptor inhibitor, atropine, suppressed SNU-C4 colon cancer cell migration; however, H508 colon cancer cell migration requires the activation of matrix metalloproteinase 7 [50, 53].

ACh binding to α7nAChR plays a functional role in the oncogenic processes [54–57]. However, published studies mostly focused on the differences in expression between non-smokers and smokers, as there is evidence that smoking increases the expression of nicotinic receptors [58, 59]. A limited number of studies on colon cancer specifically focus on the expression of α7nAChR without the influence of smoking. In lung cancer, several lines of evidence implicate the role of α7nAChR in cancer growth and metastasis [60, 61]; however, in colon cancer, less is known. Human HT-29 colon cancer cells overexpress α7nAChR, which facilitates cell proliferation, angiogenesis [62, 63], and metastasis [19, 61, 64, 65]. Our findings demonstrated no significant difference in the expression of α7nAChR at all stages of CRC. Similarly, no association between α7nAChR expression and patients’ risk of CRC and survival outcome was observed. However, α7nChR expression was strongly associated with PD-L2 and ChAT while weakly correlated with PD-L1 and M3R.

M3R signaling is reported in non-neuronal tissues, including but not limited to the colon, lung, skin, and pancreas, and ACh is shown to act as a growth factor in lung and gastric cancers [15, 17, 18, 66–68]. M3R activation increases the invasion and migration of NSCLC cells and enhances the release of interleukin (IL)-8 [24]. Furthermore, muscarinic receptors mediate the proliferation of the HT-29 cell line [69], supporting our findings. The release of ACh acting on androgen receptors promotes SNU-449 liver cancer cell invasion and migration [22]. Nicotinic receptor (nAChRs) activation by nicotine enhances LOVO and SW620 colon cancer cell invasion and metastasis [25]. Similarly, nicotine pre-treatment stimulates the activation of α9nAChR, which mediates MCF-7 and MDA-MB-231 breast cancer cell migration via the expression of epithelial-mesenchymal transition markers [70].

Most studies regarding the role of M3R in CRC were performed mainly in cell lines or animal models. Only a few studies have reported that M3R is overexpressed in human colon cancer tissues compared to normal samples. Limited studies associate the expression of M3R with different stages of CRC and clinical parameters. For instance, studies have shown that M3R is expressed in 60% of colon cancer cell lines [66, 68]. In addition, studies have reported an 8-fold increased expression of M3R in 62% of colon cancers compared to normal adjacent and normal colon epithelium [67]. In NSCLC, M3R expression is associated with tumor metastasis and poor patient prognosis [20].

The present study has illustrated that M3R is predominantly elevated at advanced stages, III + IV, compared to early stages, I + II. High levels of M3R expression correlated with a high risk of CRC and poor patient survival outcomes. In addition, M3R expression was strongly associated with PD-L1 and ChAT, whereas weakly correlated with α7nChR but not with PD-L2, suggesting that M3R might not have a functional role in PD-L2 regulation. Studies have suggested that the intracellular distribution of M3R can have detrimental effects on cancer progression [17, 51]. M3R in a normal colon epithelium is disseminated in the basolateral membrane, forming a protective barrier between the cells, the blood, and/or other cells. However, in cancer, M3R can translocate to a more dynamic environment that enhances cancer growth, such as cytoplasmic [17]. We speculate that M3R might be expressed on the surface membrane at the early stages of CRC and then translocate into the cytoplasm at the advanced stages of CRC. The activities and localization of M3R within cancer cells at various stages of CRC should be further investigated. The present study suggests that ACh might play a role in the induction of immunosuppressive molecules. This study indicates that ACh not only regulates the expression of PD-L1 and PD-L2 but could explain the inconsistency in prognostic value.

Although studies examining the role of M3R and α7nAChR in cancer have been relatively studied, there are limited studies identifying the role of ChAT in colon cancer progression. High ChAT is noted in cytoplasmic localization of H508 and Caco-5 colon cancer cells [18]. ChAT was found to be overexpressed in colon cancer specimens compared to normal colon samples [18]. Similarly, ChAT was significantly upregulated in squamous cell lung carcinoma compared to adjacent healthy specimens [51]. In the present study, the expression of ChAT correlated with advanced stages, III + IV, of CRC, compared to the early stages, I + II. In addition, high levels of ChAT were associated with a high risk of CRC and poor patient survival outcomes. Overall, the levels of ChAT were strongly correlated with the expression of M3 cholinergic receptor and moderately associated with immunosuppressive markers.

The present study findings are consistent with Kamiya et al. (2019) study in breast cancer patients demonstrating that decreased parasympathetic nerve density, determined by vesicular acetylcholine transporter expression, was associated with poor clinical outcomes and elevated levels of immune checkpoint molecules [71]. Similarly, in chemically-induced and xenograft models of breast cancer, sympathetic nerve denervation and parasympathetic neurostimulation suppressed immune checkpoint molecules, such as PD-1 and PD-L1, leading to attenuated cancer cell growth [71].

## Conclusion

The nervous and immune systems play an essential role in influencing the tumor microenvironment to promote cancer development and progression. Studies have demonstrated that modulation of the immune system by the relentless release of neurotransmitters from the nerve terminals and cancer cells can promote its growth and metastasis. However, underlying mechanisms are not understood. In addition, cancer cells’ ability to synthesize and secrete ACh promotes their proliferation, differentiation, and migration via acting as an autocrine or paracrine hormone. This study provides a novel understanding of molecular mechanisms by which the nervous system modulates cancer development and progression. Our findings show that there could be crosstalk between the expression of immunosuppressive molecules and cholinergic signaling, which may play a predictor or prognostic value in CRC patients. Therefore, revealing the interaction between the immunosuppressive and cholinergic factors in cancer is imperative for understanding CRC development and progression mechanisms.

### Electronic supplementary material

Below is the link to the electronic supplementary material.

Supplementary Material 1

## Data Availability

The datasets used and/or analyzed during the current study are available from the corresponding author on reasonable request.

## Abbreviations

4-DAMP: 1-dimethyl-4-diphenylacetoxypiperidinium iodide
a7nAChR: Alpha 7 nicotinic receptor
a9nAChR: Alpha 9 nicotinic receptor
ACh: Acetylcholine
AJCC: American Joint Committee on Cancer
AKT: Protein kinase B
ChAT: Choline acetyltransferase
CRC: Colorectal cancer
DAPI: 4′,6-diamidine-2′-phenylindole dihydrochloride
DMEM: Dulbecco’s Modified Eagle Medium
EGFR: Epidermal growth factor receptor
ERK: Extracellular-signal-regulated kinase
FACS: Fluorescence-activated cell sorting
GAPDH: Glyceraldehyde-3-phosphate dehydrogenase
HRP: Horseradish peroxidase
Hrs: Hours
IL-8: Interleukin
INF-g: Interferon gamma
M3R: Muscarinic 3 receptor
nAChRs: Nicotinic receptor
NaCl: Sodium chloride
NADPH: Nicotinamide adenine dinucleotide phosphate
NSCLC: Non-small cell lung carcinoma
PBS: Phosphate-buffered saline
PD-1: Programmed death 1
PD-L1: Programmed death ligand1
PD-L2: Programmed death ligand 2 PD-L2
PI: Propidium iodide
PI3: Phosphatidylinositol-3-OH
RIPA: Radioimmunoprecipitation assay
RPM: Revolutions per minute
RPMI: Roswell Park Memorial Institute
SDS: Sodium dodecyl sulfate
SEM: Standard error mean
STAT3: Signal transducer and activator of transcription 3
Th1: T helper types 1
Th2: T helper types 2
WST-1: Water-soluble tetrazolium-1
