# Supplementary material for: Cholinergic signaling influences the expression of immune checkpoint inhibitors, PD-L1 and PD-L2, and tumor hallmarks in human colorectal cancer tissues and cell lines
Source: BMC Cancer. 2023 Oct 12;23:971. doi: 10.1186/s12885-023-11410-3 (PMC10568879; doi:10.1186/s12885-023-11410-3)
Supplement: Supplementary file 1 — Supplementary Material 1 [file 12885_2023_11410_MOESM1_ESM.pdf]

Western blot bands for LIM-2405, and HT-29 ran on the same blot with a well separating each cell line are shown. PD-L1 expression was evaluated in cells treated with C: control, A: atropine, 4-D: 4-DAMP.

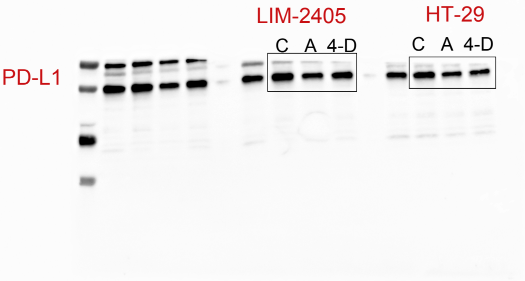

LIM-2405
HT-29
C
A
4-D
C
A
4-D
PD-L1

Western blot bands for LIM-2405, and HT-29 ran on the same blot with a well separating each cell line are shown. PD-L2 expression was evaluated in cells treated with C: control, A: atropine, 4-D: 4-DAMP.

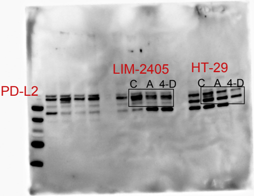
PD-L2
LIM-2405
C A 4-D
HT-29
C A 4-D

Western blot bands for LIM-2405, and HT-29 ran on the same blot with a well separating each cell line are shown. GAPDH expression was evaluated in cells treated with C: control, A: atropine, 4-D: 4-DAMP.

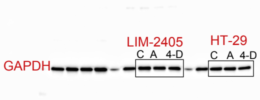
LIM-2405
HT-29
C A 4-D
C A 4-D
GAPDH

Western blot bands for LIM-2405, and HT-29 ran on the same blot with a well separating each cell line are shown. M3R expression was evaluated in cells treated with C: control, A: atropine, 4-D: 4-DAMP.

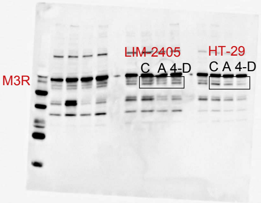

M3R
LIM-2405
C A 4-D
HT-29
C A 4-D

Western blot bands for LIM-2405, and HT-29 ran on the same blot with a well separating each cell line are shown. ChAT expression was evaluated in cells treated with C: control, A: atropine, 4-D: 4-DAMP.

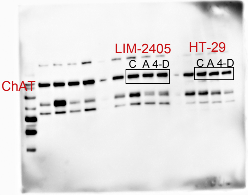
ChAT
LIM-2405
C A 4-D
HT-29
C A 4-D

Western blot bands for LIM-2405, and HT-29 ran on the same blot with a well separating each cell line are shown. EGFR expression was evaluated in cells treated with C: control, A: atropine, 4-D: 4-DAMP.

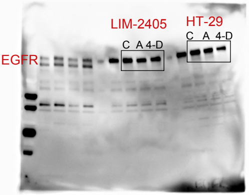

LIM-2405
HT-29
C A 4-D
C A 4-D
EGFR

Western blot bands for LIM-2405, and HT-29 ran on the same blot with a well separating each cell line are shown. pERK expression was evaluated in cells treated with C: control, A: atropine, 4-D: 4-DAMP.

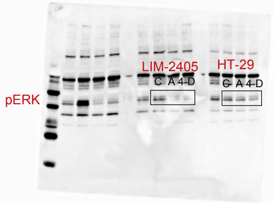
pERK
LIM-2405
C A 4-D
HT-29
C A 4-D

Western blot bands for LIM-2405, and HT-29 ran on the same blot with a well separating each cell line are shown. pSTAT expression was evaluated in cells treated with C: control, A: atropine, 4-D: 4-DAMP.

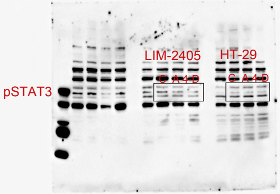
pSTAT3
LIM-2405
HT-29
C A 4-D
C A 4-D
